# Analysis of leg bone lengths from whole-body dual-energy X-ray absorptiometry in the UK biobank

**DOI:** 10.1093/bjrai/ubag014

**Published:** 2026-08-28

**Authors:** Faten Alomar, Junkai Wang, Timothy F Cootes

**Affiliations:** Department of Computer Science, College of Computer and Information Sciences, King Saud University, Riyadh 12372, Saudi Arabia; Department of Computer Science, The University of Manchester, Manchester M13 9PL, United Kingdom; Department of Computer Science, The University of Manchester, Manchester M13 9PL, United Kingdom; Division of Informatics, Imaging and Data Science, The University of Manchester, Manchester M13 9PT, United Kingdom

**Keywords:** femur, tibia, DXA images, UK Biobank

## Abstract

**Objectives:**

To calculate the distributions of leg bone lengths (as measured from dual-energy X-ray absorptiometry [DXA] images) and their relationship with height. Estimating human height from long bone measurements is widely used in forensic and biological anthropology.

**Methods:**

An automated system was developed to find the outlines of the femur and tibia in DXA images from the UK Biobank. These were used to measure the length of the bones. Images with implants, severe abnormalities, or missing data were excluded. Relationships between lengths and with height were estimated using linear regression.

**Results:**

Of the 73 405 available DXA images, we successfully measured both femurs and tibia in 66 730 cases (91%). The lengths of femurs and tibias for males and females each show normal distributions, with left and right being very similar. The mean absolute difference between measurements at 2 time points was below 1.8 mm (femur), 2.3 mm (tibia), showing the system is repeatable. Linear regression gives the relationships between individual bone lengths and between them and height.

**Conclusions:**

The developed software system and the resulting analysis provide promising insights into bone geometry using DXA imaging, enabling large-scale, precise bone measurements. These models are relevant for predicting height, a subject of interest in archaeology and forensic science.

**Advances in knowledge:**

This is the largest study of the distributions of leg bone lengths and their relationship to height that we are aware of.

## Introduction

Whole-body dual-energy X-ray absorptiometry (DXA) images have been collected from 68 652 participants in the UK Biobank imaging study. This enables the study of bone length distributions in large populations. We have developed a system that automatically locates femur and tibia outlines in images. We have used this to study the distributions of bone length across a population and investigate the relationship between bone length and stature (height).

Estimating height from long-bone lengths is a well-established task in forensic science and biological anthropology, and it can also support clinical assessments such as calculating BMI in hospitalized or bedridden patients who cannot stand for direct measurement. Because body proportions vary across human populations, stature inference must rely on formulae derived from the specific population of interest, and numerous studies have shown that predictive accuracy is affected by these population differences.^1–3^ Although several regression formulae exist—based on measurements obtained from autopsied individuals^4^^,^^5^ or skeletal remains^1^^,^^6^ most prior work relies on relatively small samples and data from deceased bodies, with only a few studies involving living participants.^7^ Beyond stature estimation, analysis of femur and tibia shapes and orientations also plays a key role in early detection of musculoskeletal disorders such as osteoarthritis^8^^,^^9^ and bone lengths form an important component of optimal surgical planning^10^ and biomechanical research.^11–13^ Together, these applications highlight the broader clinical and scientific value of accurately characterizing long-bone dimensions in living populations.

This article describes the automated bone outline location system and evaluates its performance. We show that male and female bone lengths are normally distributed, with males having a longer bones but with a similar SD. We demonstrate that most subjects have similar left and right bone lengths. We present the distributions of various measurements and relationships with height. This is the largest study of the relationship between height and bone lengths that we are aware of.

### Previous work

Anthropologists and forensic scientists studying skeletal remains often estimate height (stature) from bones.

Trotter and Gleser^1^ used measurements of living height and bone lengths after skeletonization to estimate height based on long limb bone length. They presented regression equations for several ethnic groups.

Aitken^14^ set a benchmark by establishing normative values for femoral and tibial lengths from full-length radiographs of standing subjects.

In forensic anthropology, Banyeh et al^15^ explored sex and height estimation from percutaneous lengths of the ulna and tibia. They showed the importance of considering specific demographic characteristics in height estimation. Saco-Ledo et al^7^ examined the relationship between tibial length and stature.

Brits et al^16^ developed regression formulas for height estimation from the femur and tibia lengths measured from magnetic resonance imaging (MRI).

Mulenga^17^ conducted a cross-sectional study to ascertain human height using femur length.

Moitra^18^ explored the correlation between height and tibial length.

Duyar and Pelin^19^ looked at the relationship between height and tibia length and suggested using separate relationships for short and tall subjects to enhance accuracy.

Gualdi-Russo et al^20^ and Bidmos and Brits^21^ looked at bone lengths and height.

We summarize the prediction equations from some of these works in Table 5.

## Methods

### Data

We have analyzed DXA images from the UK biobank (https://www.ukbiobank.ac.uk). UK Biobank is a comprehensive biomedical database and research tool that houses genetic, lifestyle, and health data and biological samples from 500 000 people in the United Kingdom. All data are anonymized to protect privacy.^22^ The UK Biobank Imaging Study has collected whole-body bone images (Figure 1) from 100 000 of these subjects.

**Figure 1:**
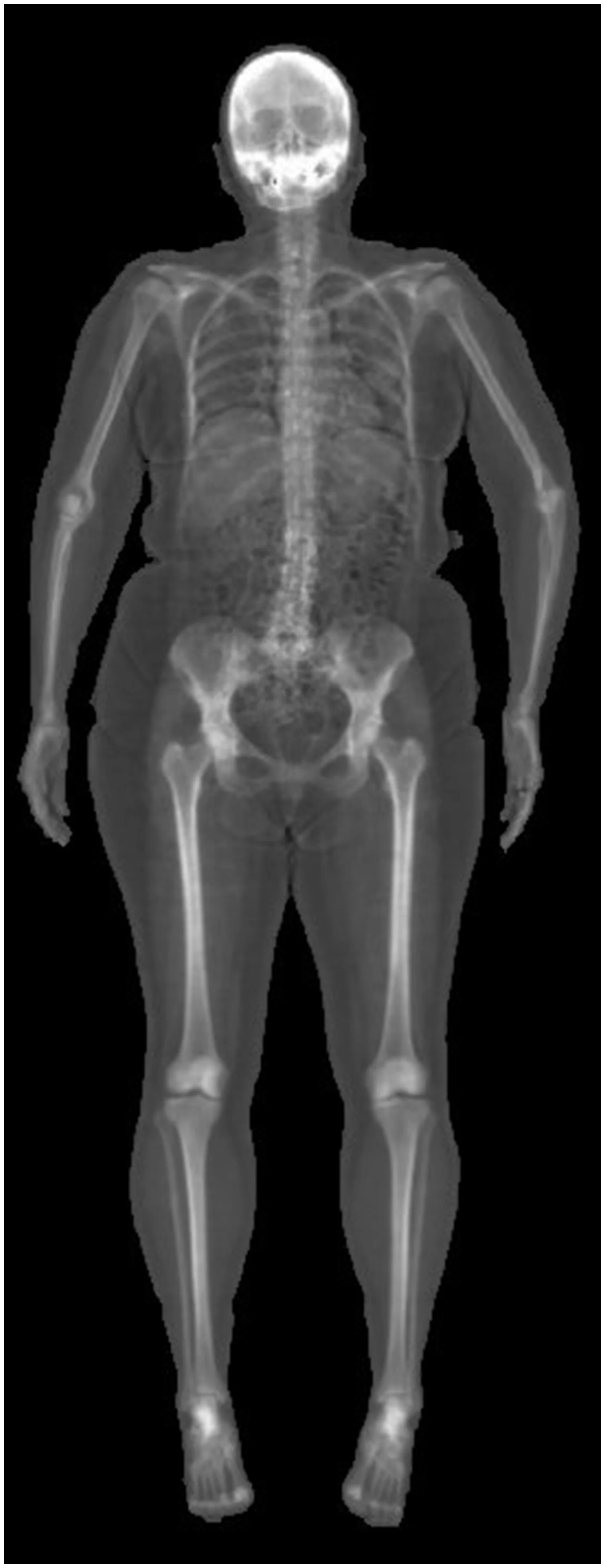
Example bone DXA image from UK Biobank.

The UK Biobank records many measurements of each subject, including sex, age, weight, and height at the time of imaging. The whole-body images include variations in patient positioning. Some images show hip or knee implants.

Each imaging session produces 2 whole-body images, one showing the bones and the other showing the tissue.

#### Annotation

The first author manually annotated points at the ends of the femur and tibia on 1000 images (Figure 2) and detailed outlines of the femur and tibia on 500 images (Figures 3 and 4).

**Figure 2:**
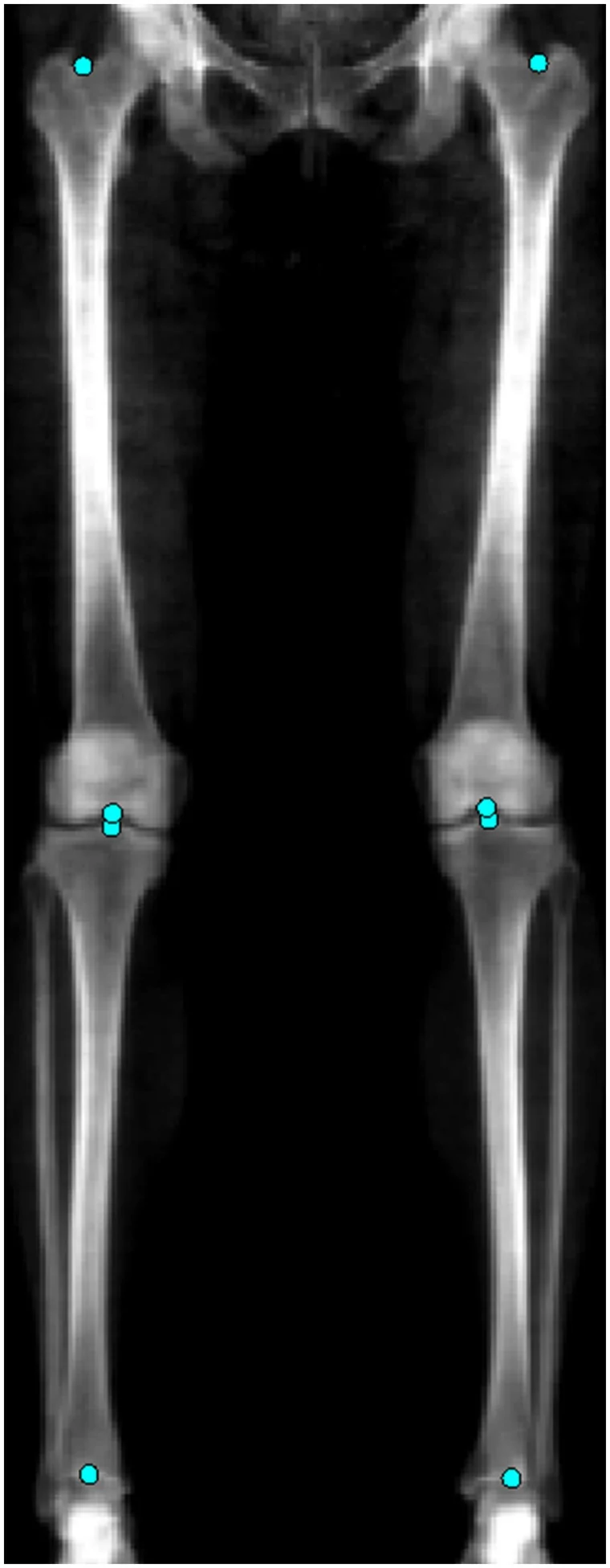
Points marking the ends of the femurs and tibias.

**Figure 3:**
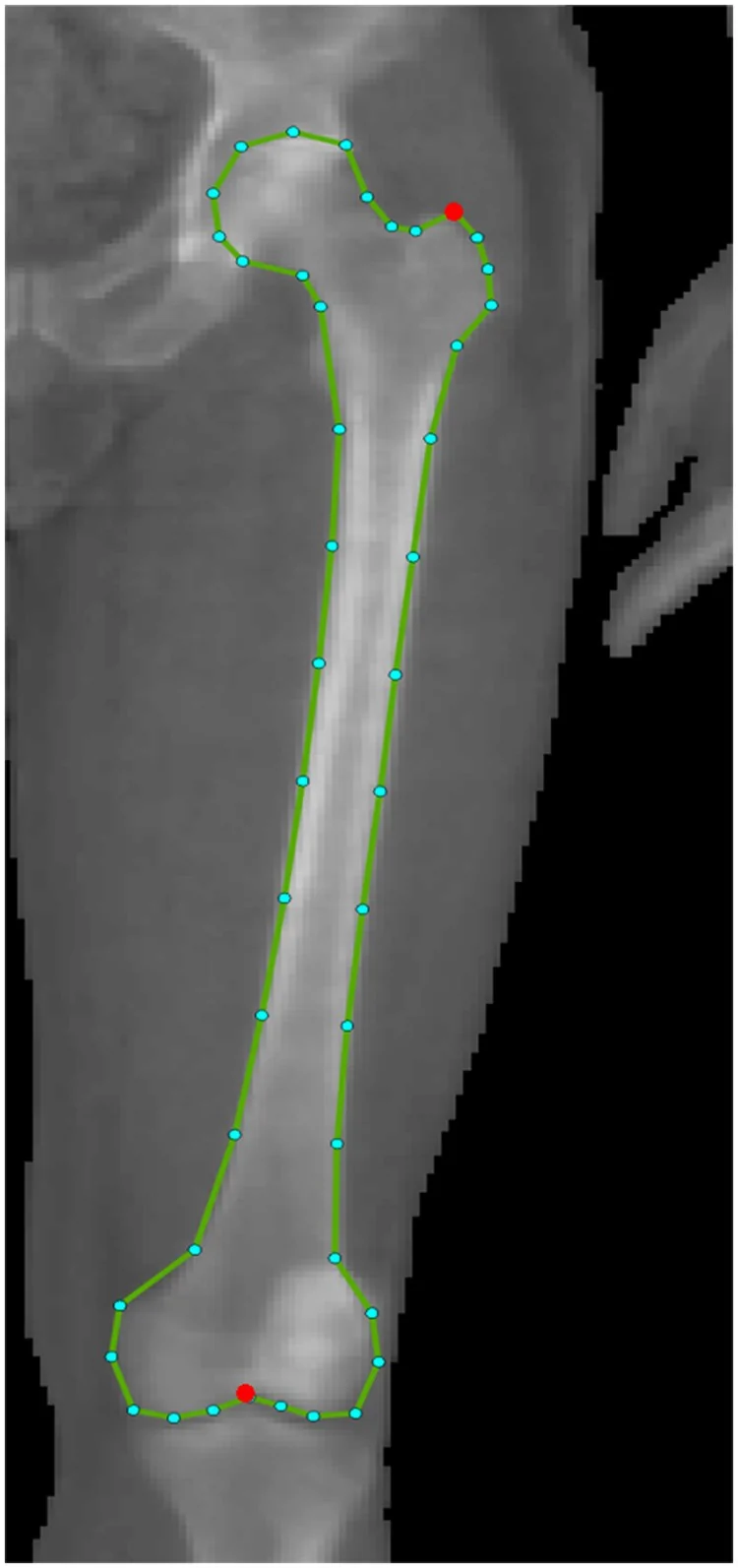
Constructing the femur annotation (43 points around outline). The red points are used to calculate the length.

**Figure 4:**
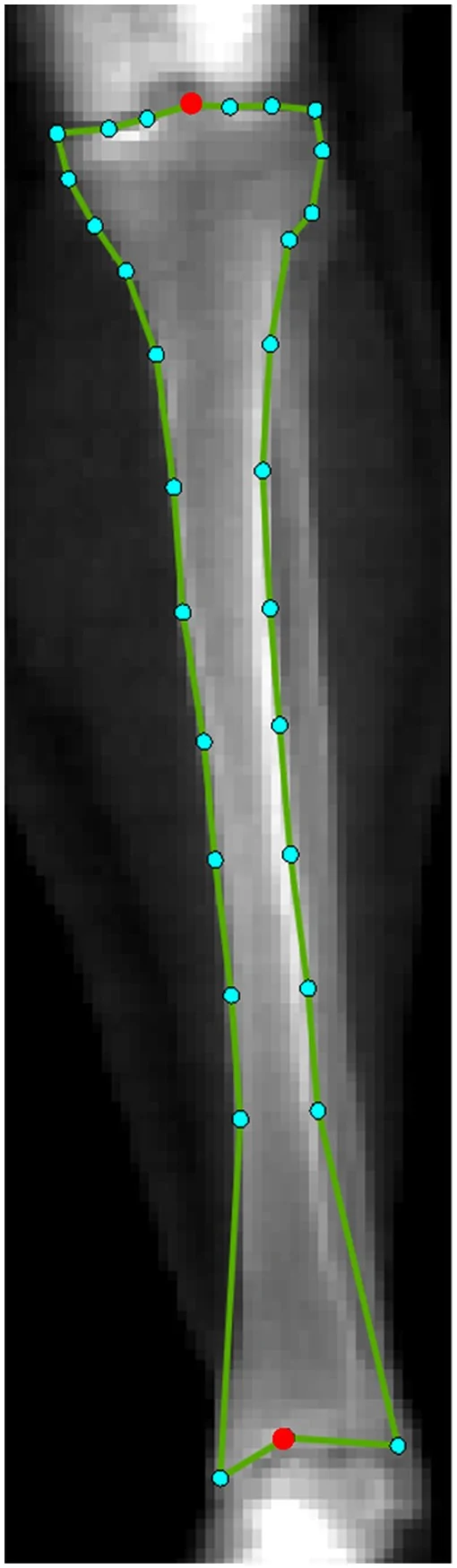
Constructing the tibia annotation (30 points around outline). The red points are used to calculate the length.

##### Full shape

We created detailed annotations for 500 images representing the bones’ full outline. We used existing models of the proximal femur from Faber et al^23^ and the knee from Beynon et al.^24^ These models were trained on higher-resolution DXA images of the hip and femur but were found to work well when applied to lower-resolution whole-body images. They were applied to all 500 images. The results were visually checked by the first author and corrected where necessary. The first author also manually annotated points equally spaced along either side of the shaft of each bone.

The detailed knee and hip models produced points with separation of less than the pixel size (1.9 × 1.9 mm) in the whole-body images, we selected a subset of the points to get a final annotation—see Figures 3 and 4.

The final points were used to construct statistical shape models^25^ and models capable of searching for the landmarks on new images (Random Forest Regression-voting Constrained Local Models, RFCLMs^26^). A global search was applied to find the approximate positions of the ends of the bones (Figure 2). These were then refined using a local search. The resulting points were used to initialize a final local search using the femur and tibia models to find the outlines of the bones (the models will be made available for others to use via the www.bone-finder.com site. Length measurements will be uploaded to UK Biobank).

Although the 2 red end-points in Figures 3 and 4 are used to calculate bone length, identifying these end-points accurately requires locating additional anatomical landmarks along the femur and tibia. The full sets of 43 and 30 points provide a more robust representation of bone shape, which helps the statistical shape model reliably determine the positions of the true proximal and distal end-points.

To evaluate the system’s accuracy, we conducted 5-fold cross-validation on the 500 manually annotated images used during training. The data were randomly split into 5 groups. For each group of 100, we trained the model on the other 400 images and ran it on the images in the unused group. We compared the points found by the automated search with the ground truth manually checked annotations. By cycling through all 5 groups all images were tested using a model that did not include that image in its training set. We quantified the accuracy of a model on a single image as the mean of the distances between each point found by the model and the equivalent manually annotated point. We report the mean, median, and 95%ile of this value over the whole set of images (Table 1). The accuracy that can be achieved is limited by the pixel size (about 1.9 mm × 1.9 mm). To measure the lengths of the bones we calculated the Euclidean distance between the 2 landmarks at either end (highlighted in red in Figures 3 and 4).

**Table 1:** Accuracy of search (mean distance from ground truth points, mm).

	Both ends		Full shape	
	Femur	Tibia	Femur	Tibia
	2 pts	2 pts	43 pts	30 pts
**Median distance (mm)**	1.9	1.8	3.7	3.7
**Mean (mm)**	2.2	2.4	3.8	4.4
**95%ile (mm)**	4.8	4.7	5.9	8.0

To estimate the repeatability of the length measurements, we used images from participants who had a second set of DXA images taken about 2 years after the baseline images. If we assume that there will be only very small changes in bone length over this time period, the majority of any discrepancies between lengths will be due to the inaccuracy of the measurements.

## Results

### Accuracy of point location

We used the RFCLM^27^^,^^28^ framework to locate points on new images.

### Annotating all images

We applied the RFCLM approach to locate key points on 73 405 whole-body bone DXA images and implemented several filtering steps to ensure data quality. The following images were excluded: incomplete or corrupted images, subjects for whom no bone images were available, those showing hip implants, and those with knee implants or leg metalwork.

The number of cases remaining after these steps is shown in Table 2. In total, 66 730 images remained after all exclusions.

Knee implants were identified using a CNN classifier trained to distinguish normal knees from those with implants.

**Table 2:** Numbers of images containing valid data after exclusions.

	Number of images
**Initial images**	73 405
**Remove incomplete or corrupted images**	73 164
**Remove those without bone images**	73 123
**Remove those with hip implants**	72 924
**Remove those with knee implants or metalwork**	71 451
**Remove abnormal cases**	71 384
**Remove second visits**	66 730

The automatic search system produces a quality-of-fit score for each bone. Results were ranked using this score and the worst cases were manually inspected by the lead author. We identified search failures, implants, and severely abnormal bones during this process. Cases in which the search failed were manually corrected. Results were examined in rank order (worst fit first) and continued until the density of rejects fell below about 1 in 100 images. During this process the points on 1920 femur and 1896 tibia were manually corrected.

### Repeatability of length measurements

Table 3 shows the statistics of the differences between the lengths at each time point. The mean absolute difference is less than one pixel (1.8 mm) for the femur, showing that the system is producing repeatable estimates. The differences for the tibias are slightly larger, perhaps because the distal end is harder to locate reliably.

**Table 3:** Mean, SD, mean absolute difference, and 95%ile absolute difference (mm) between lengths at 2 time-points for 4673 subjects.

Bone	Mean	SD	MAD	95%ile AD
**Left femur**	0.1	1.8	1.3	3.8
**Right femur**	0.1	2.0	1.3	3.6
**Left tibia**	0.0	3.9	2.1	5.6
**Right tibia**	0.1	4.8	2.3	5.8

### Bone lengths

Table 4 shows the mean and SD for femur and tibia bone lengths by sex.

**Table 4:** Lengths of bones for 32 043 male and 34 687 female subjects.

	Male		Female	
	Femur	Tibia	Femur	Tibia
**Mean (mm)**	445	377	411	345
**SD (mm)**	22	21	21	19

**Table 5:** Summary of regression equations for predicting femur and tibia lengths (in mm).

Study	Group	No. subjects	Height		Femur		Tibia		**Height from femur** ^a^		**Height from tibia** ^b^		Height from femur and tibia^c^		
			Mean	SD	Mean	SD	Mean	SD	*C*_F_	*C*_0_	*C*_T_	*C*_0_	*C*_F_	*C*_T_	*C*_0_
**Male**															
**UK Biobank (ours)**	UK (90% White)	32 043	1766	66	445	22	377	21	2.57	621	2.68	756	1.47	1.30	621
**Sebastian Simon^29^**	Austrians	1674	1771	74	471	43	378	36	2.08	775	2.42	864	1.21	1.21	735
**Trotter and Gleser^1^**	White American	4672	1740	66	471	24	384	22	2.28	671	2.42	816	1.14	1.37	683
**Trotter and Gleser^1^**	Black American	577	1734	66	483	25	403	24	2.10	720	2.19	854	1.33	0.95	714
**Gonzalo Saco-Ledo^7^**	Spanish	249	1785	68	–	–	405	24	–	–	3.29	480	–	–	–
**Moitra^18^**	West Bengal	200	1676	71	–	–	361	29	–	–	1.24	1230	–	–	–
**Gualdi-Russo^20^**	Mediterranean	219	1782	70	–	–	402	29	–	–	1.66	1114	–	–	–
**Izzet Duyar^19^**	Turkish	231	1746	91	–	–	390	30	–	–	2.74	679	–	–	–
**Female**															
**UK Biobank (ours)**	UK (90% White)	34 687	1634	61	411	21	345	19	2.55	584	2.70	702	1.56	1.21	577
**Sebastian Simon^29^**	Austrians	2526	1634	65	431	43	350	28	1.90	798	2.30	842	1.12	1.12	755
**Moitra^18^**	West Bengal	200	1574	56	–	–	335	36	–	–	0.34	1460	–	–	–
**Iwao Hasegawa ^30^**	Japanese	342	1574	52	412	18	330	17	2.33	614	2.47	758	1.21	1.47	589

a Height = *C*_F_ × femur + *C*_0_.

b Height = *C*_T_ × tibia + *C*_0_.

c Height = *C*_F_ × femur + *C*_T_ × tibia + *C*_0_.

The normalized histograms in Figures 5 and 6 show that the distributions are almost perfect Gaussians. On average, femurs from males are 36 mm longer than those from females, and tibias are 32 mm longer. We found that the lengths of the femur and tibia were highly symmetric (left and right were very close in length).

**Figure 5:**
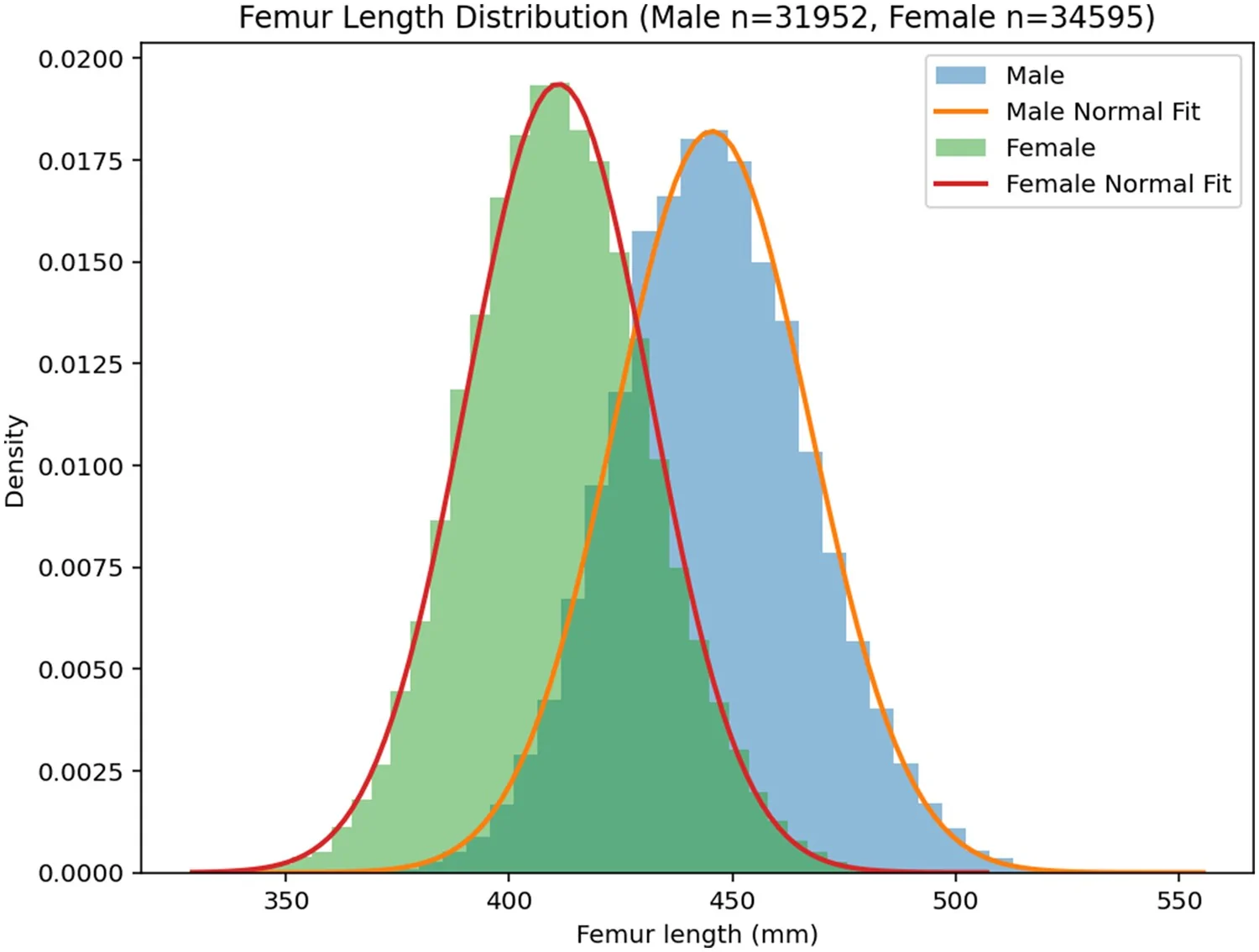
Distribution of femur length (mm) for 32 043 males and 34 687 female subjects.

**Figure 6:**
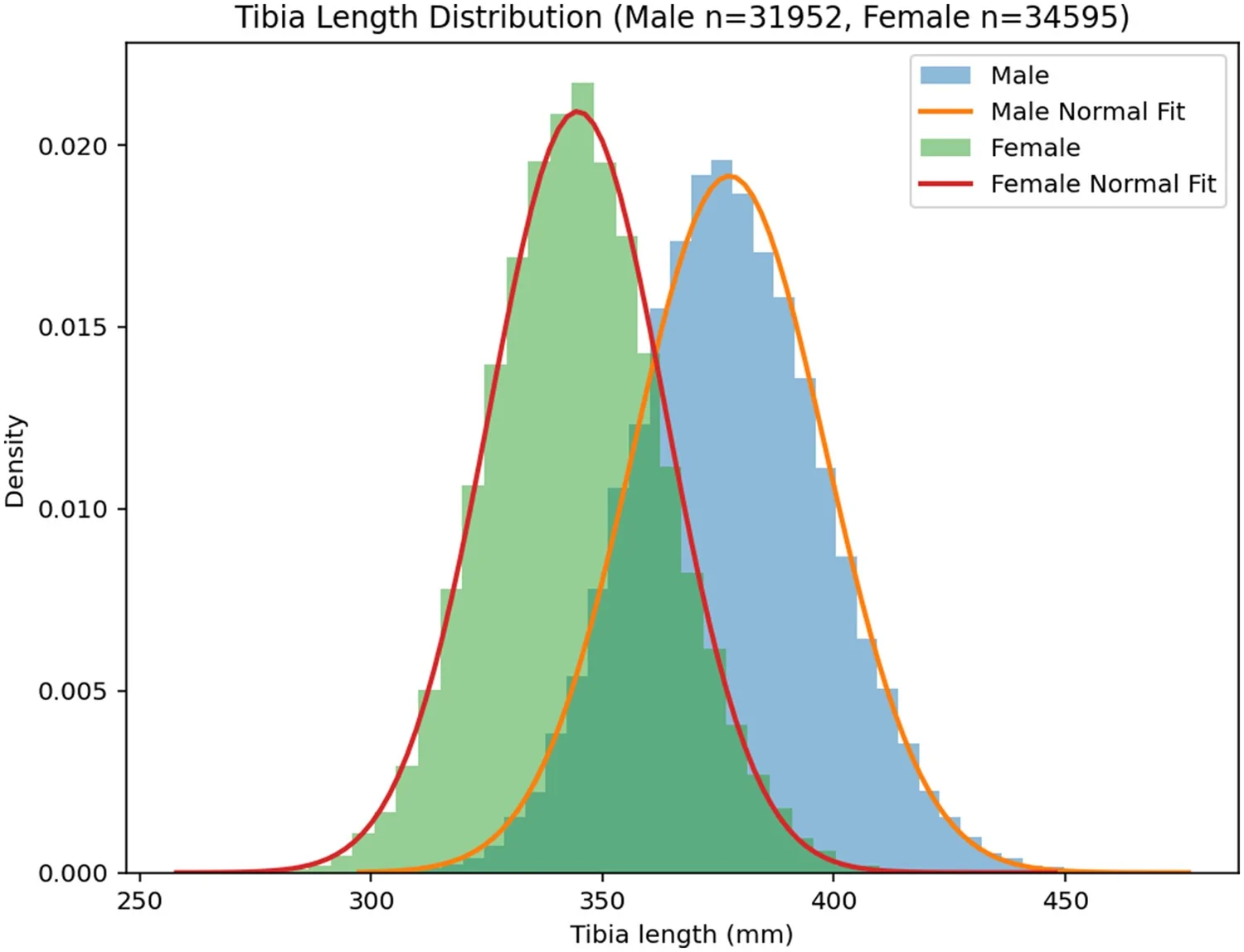
Distribution of tibia length (mm) from 32 043 male and 34 687 female subjects.

#### Relationship between femur and tibia lengths

We examined the relationship between femur and tibia lengths using the 66 730 images in which all bones were found. For each individual, we took the average of the left and right bones as the best estimate of the bone length. Figure 7 shows the scatter of femur length against the tibia length for males and females.

**Figure 7:**
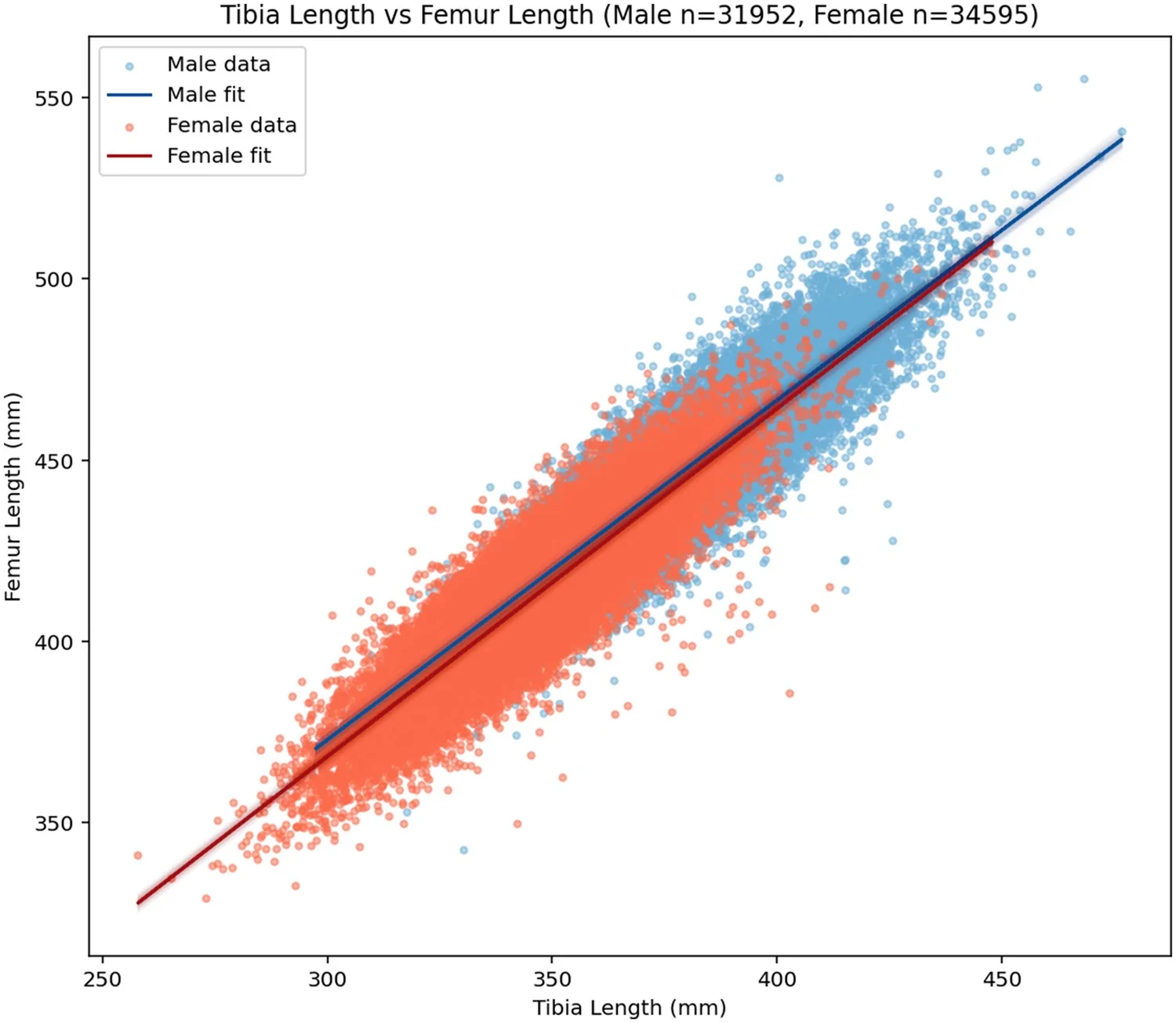
Femur versus tibia length (32 043 male and 34 687 female subjects).

#### Relationship between height and bone lengths

The height of each subject was recorded around the time they were imaged. We used linear regression to estimate the relationship between individual and combined bone lengths and height.

Figure 8 shows the relationships between height and femur length for each sex. The best-fitting linear equations for males and females are:


(1) $$\mathrm{Height}(\mathrm{Male})=2.57L_{\mathrm{femur}}+621\;\mathrm{mm}$$

(2) $$\mathrm{Height}(\mathrm{Female})=2.55L_{\mathrm{femur}}+584\;\mathrm{mm}$$

**Figure 8:**
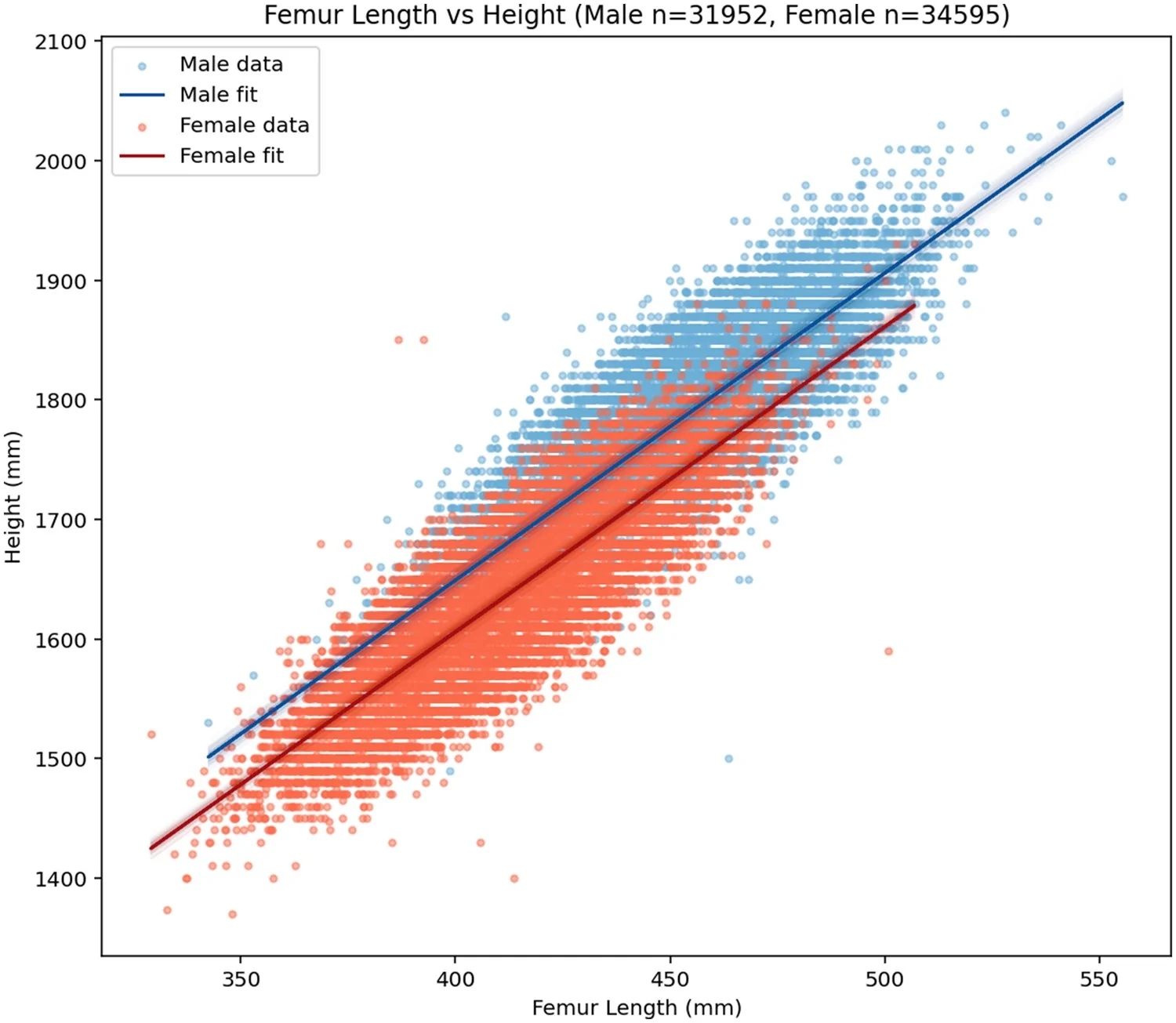
Frequency plot and best fitting line showing relationship between height and femur length (32 043 male and 34 687 female subjects).

For males, $R^{2}$ = 0.73, RMS error = 34 mm, 95% confidence intervals for slope: 2.55-2.59, and 95% CI intercept: 614-629 mm. For females, $R^{2}$ = 0.73, RMSE = 32 mm, 95% CI slope: 2.54-2.57, and 95% CI intercept: 578-591 mm.

Figure 9 shows the relationships between height and tibia length for each sex. The best-fitting linear equations for males and females, respectively, were as follows: males, $R^{2}$ = 0.72, RMSE = 35 mm, 95% CI slope: 2.66-2.70 and 95% CI intercept: 749-763 mm; females: $R^{2}$ = 0.71, RMSE = 33 mm, 95% CI slope: 2.69-2.72, and 95% CI intercept: 696-708 mm.


(3) $$\text{Height}(\text{Male})=2{.68L}_{\text{tibia}}+756\;\text{mm}$$

(4) $$\text{Height}(\text{Female})=2{.70L}_{\text{tibia}}+702\;\text{mm}$$

**Figure 9:**
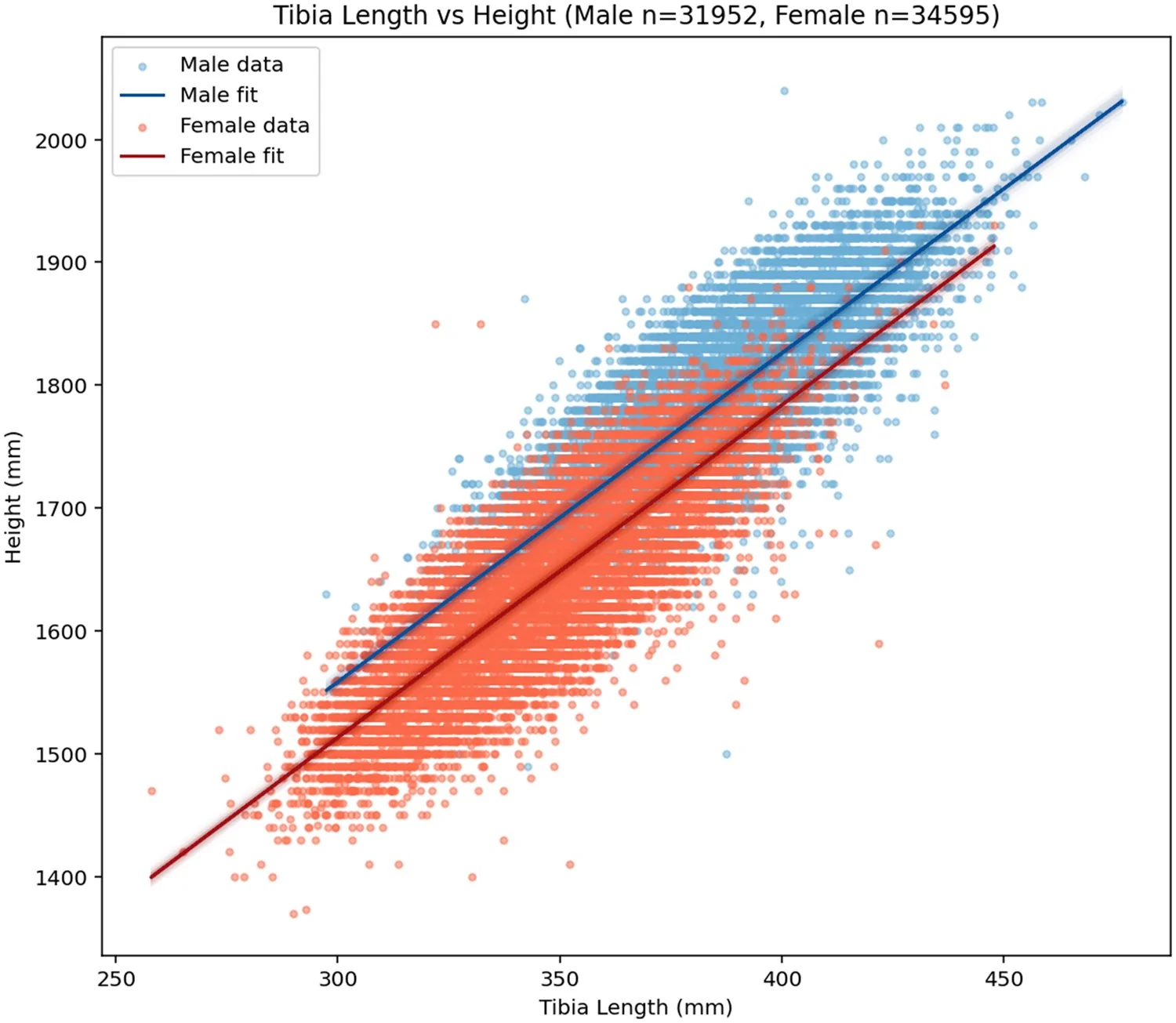
Frequency plot and best fitting line showing relationship between height and tibia length (32 043 male and 34 687 female subjects).

We used linear regression to estimate height from the femur and tibia lengths together:


(5) $$\begin{array}{c} \text{Height}(\text{Male})=1{.47L}_{\text{femur}}+1{.30L}_{\text{tibia}}+621\;\text{mm} \end{array}$$

(6) $$\text{Height}(\text{Female})=1{.56L}_{\text{femur}}+1{.21L}_{\text{tibia}}+577\;\text{mm}$$

Figure 10 shows the effectiveness of the best fitting regression model for female subjects, with $R^{2}$ = 0.76, RMSE = 30mm, 95% CI femur slope: 1.56-1.57, 95% CI tibia slope: 1.20-1.21, and 95% CI intercept: 567-586 mm. For males, the $R^{2}$ = 0.77, RMSE = 32 mm, 95% CI femur slope: 1.47-1.48, 95% CI tibia slope: 1.29-1.30, and 95% CI intercept: 611-632 mm.

**Figure 10:**
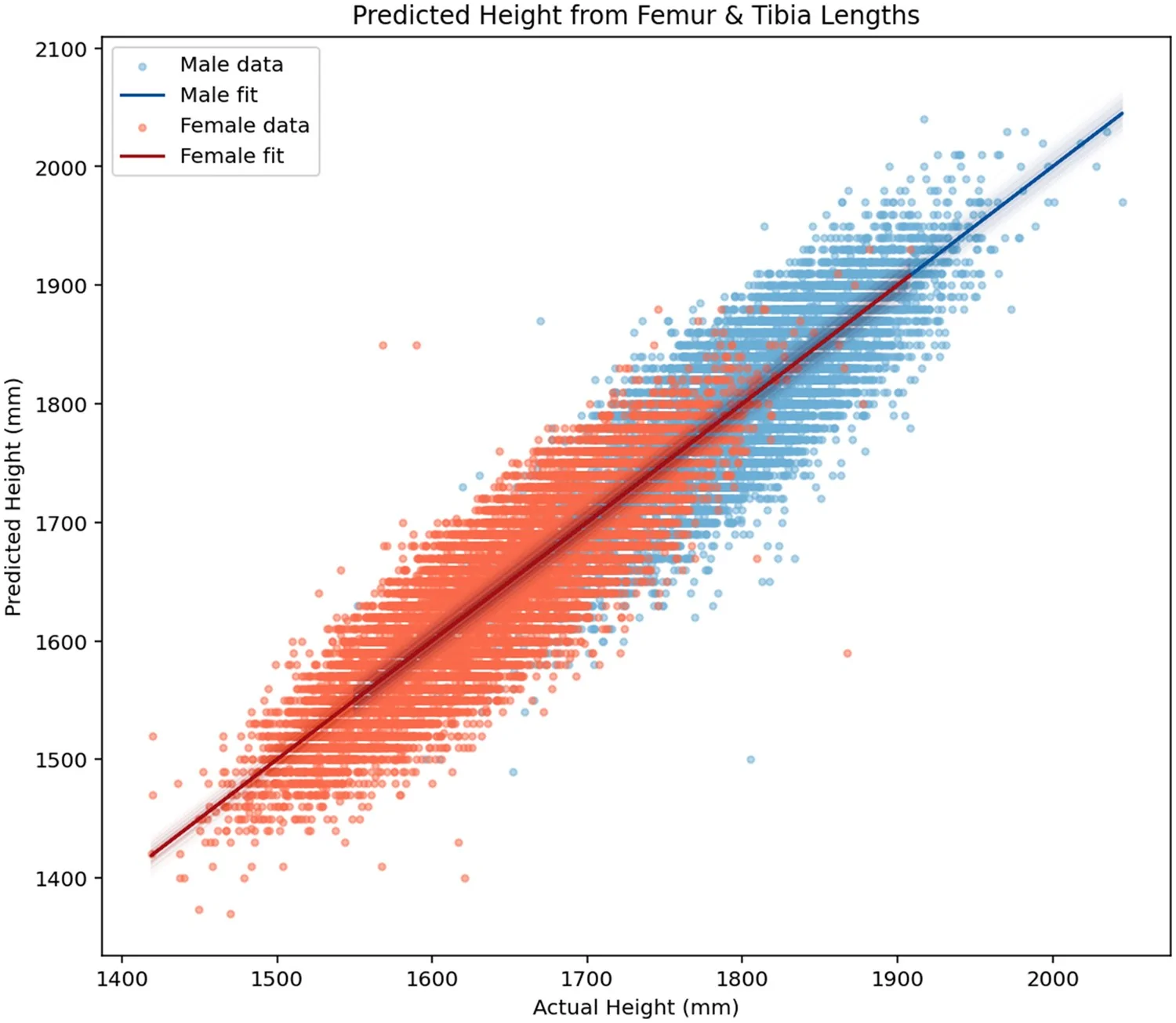
Frequency plot and best fitting line showing relationships between height and average leg bone lengths (32 043 male and 34 687 female subjects).

Figures 7-10 show the density plots for females. Those for males have a very similar form but with different equation coefficients.

To quantify potential sex dimorphism in body proportions, we compared femur-to-height and tibia-to-height ratios between males and females. Femur-to-height ratios were virtually identical in males and females (0.252:1 vs 0.251:1), although the large sample size produced a statistically significant difference in its hypothesis test (t=13.95, $P=3.5\times 10^{-44}$). The effect size was very small (Cohen’s d=0.11), indicating negligible dimorphism. In contrast, tibia-to-height ratios showed a slightly larger sex difference (males: 0.214:1; females: 0.211:1; t=55.77, $P<10^{-300}$). Together, these results show that sex differences in long-bone scaling exist but are modest, supporting the use of sex-specific models.

## Discussion

### Limitations

We measured the lengths of the bones, which are 3D structures, from 2D images. There will be some inaccuracy because the bones are not parallel to the imaging plane. However, in most cases, the error due to this will be small. The pixel sizes (1.9×1.9 mm) also limit the accuracy. We did not have access to an independent measurement of the bone lengths, and so could not compare our measurements with alternatives. It may be possible to improve the accuracy with which points are found by using modern deep-learning techniques. However, the many subjects involved will help to overcome some of the uncertainty in estimates of the lengths introduced by errors in the search. Different studies use different ways of measuring the length (different reference points), so are not all directly comparable. Most studies measure femur and tibia length using the most extreme points at either end, identified manually or through predefined anatomical landmarks. These points can be hard to locate repeatably, so we have chosen points that could be identified reliably. For instance, the top of the femur head is often hard to locate because of occlusion by the pelvis. Instead, we use the top of the greater trochanter. Thus, our results may be slightly shorter to those that measure to the femur head.

Table 5 presents the relationships between height and femur/tibia lengths reported in this and previous studies. This study included far more subjects than the previous largest study. There are differences between the coefficients for males and females across different national/ethnic groups.

Femur length showed no meaningful association with age in either males or females. Correlation coefficients were close to 0 (r=−0.03), and regression slopes indicated minimal change in femur length, suggesting that femur length remains largely stable with increasing age. In contrast, femur length demonstrated a moderate positive association with body weight in both sexes. The relationship was stronger in males (r=0.35) than in females (r=0.27), indicating that individuals with higher body weight tend to have longer bones. The magnitude of this association was modest and substantially weaker than the relationships observed between femur length and overall height.

This study looked at estimating the lengths of bones from DXA images in the UK Biobank. We demonstrated the repeatability of the system by comparing left with right and by looking at measurements from 2 different time-points. We have used the measurements to explore the relationship between long-bone lengths and height. In future work, we aim to explore the relationships between genetics and bone lengths, and how bone lengths affect the risks and progression of musculoskeletal diseases such as osteoarthritis.
